# Supplementary material for: Deficits in integrative NMDA receptors caused by Grin1 disruption can be rescued in adulthood
Source: Neuropsychopharmacology. 2023 Jun 22;48(12):1742–51. doi: 10.1038/s41386-023-01619-y (PMC10579298; doi:10.1038/s41386-023-01619-y)
Supplement: Supplementary file 1 — Supplemental Figures, Tables, and Methods [file 41386_2023_1619_MOESM1_ESM.docx]

**Supplemental Tables, Figures, and Methods**

**Deficits in integrative NMDA receptors caused by *Grin1* disruption can be rescued in adulthood**

Sridevi Venkatesan^1*^, Mary A. Binko^1*#^, Catharine A. Mielnik^2^, Amy J. Ramsey^1,2^, Evelyn K. Lambe^1,3,4^

- **Supplemental Tables S1, S2, S3**
- **Supplemental Figures S1, S2, S3**
- **Supplemental Methods**

**Supplemental Tables**

| **Cortical NMDARs in *Grin1* knockdown mice** | | **Grin1KD**  **(% WT)** | **Change** |
| --- | --- | --- | --- |
| **Protein measurements** | | | |
| 1 | Western blot [1] |  |  |
|  | Total GluN1 | 5-9% | **↓↓↓↓** |
|  | Total GluN2A | 14 ± 3% | ↓↓↓↓ |
|  | Total GluN2B | 12 ± 1% | ↓↓↓↓ |
|  |  |  |  |
| 2 | Synaptic GluN1  (PSD95 immunoprecipitation & mass spectrometry) [2] | 32 ± 3% | ↓↓ |
| **mRNA measurements** | | | |
| 1 | *Grin1* Fluorescent in situ hybridization [2] | Qualitative | ↓↓ |
| 2 | RNAseq (cortex) [3] | *(log_2_ fold change)* |  |
|  | *Grin1* | -1.285 | ↓↓ |
|  | *Grin2a* | 0 | **−** |
|  | *Grin2b* | 0 | **−** |

**Supplemental Table S1.**

**Molecular characterization of NMDAR subunits in *Grin1* knockdown mice.** Cortical protein and mRNA levels of GluN1 and GluN2 subunits measured in Grin1 KD mice in previous studies are reported here. (↓ decrease, **−** no change). Synaptic GluN1 protein levels are better preserved (32% of wildtype) compared to total GluN1 protein (5-9% of wildtype). From the following references: *[1] Ramsey et al., Neuropsychopharm, 2008; [2] Mielnik et al., Molecular Psychiatry, 2021. [3] Mielnik, C. Inducible Rescue of N-Methyl-D-Aspartate Receptor Deficiency to Study Brain Plasticity. Thesis. 2017.*

| **Property** | **WT**  *n* = 20 | ***Grin1*KD**  *n* = 25 | **unpaired *t* test** |
| --- | --- | --- | --- |
| Resting Potential (mV) | -82 ± 1 | -80 ± 1 | *t_43_* = 1.84, *P* = 0.08 |
| Input Resistance (MΩ) | 95 ± 6 | 95 ± 88 | *t_43_* = 0.08, *P* = 0.93 |
| Capacitance (pF) | 172 ± 7 | 162 ± 5 | *t_43_* = 1.13, *P* = 0.26 |
| Spike Amplitude (mV) | 88 ± 1 | 89 ± 1 | *t_43_* = 0.73, *P* = 0.47 |
| Rheobase (pA) | 127 ± 12 | 118 ± 11 | *t_40_* = 0.17, *P* = 0.86 |

**Supplemental Table S2.**

**Knockdown of *Grin1* does not significantly alter neuronal properties.**

Intrinsic electrophysiological properties of prefrontal layer 5 pyramidal neurons from littermate WT and *Grin1*KD mice. Data shown as mean ± SEM.

| **Property** | **WT**  *n* = 10 | ***Grin1*KD**  *n = 13* | ***Grin1*rescue**  *n = 13* | **One-way ANOVA** |
| --- | --- | --- | --- | --- |
| Resting Potential (mV) | -86 ± 1 | -83 ± 2 | -83 ± 2 | F _(2, 33)_ = 0.70, *P* =0.50 |
| Input Resistance (MΩ) | 131±15 | 132±15 | 125±10 | F _(2, 33)_ = 0.08, *P* =0.93 |
| Capacitance (pF) | 103 ± 9 | 124 ± 5 | 112 ± 7 | F _(2, 33)_ = 2.28, *P* =0.12 |
| Spike Amplitude (mV) | 81 ± 2 | 76 ± 2 | 76 ± 2 | F _(2, 33)_ = 1.10, *P* =0.35 |
| Rheobase (pA) | 95 ± 18 | 98 ± 21 | 89 ± 10 | F _(2, 32)_ = 0.08, *P* =0.92 |

**Supplemental Table S3.**

**Adult rescue of *Grin1* did not significantly alter neuronal intrinsic properties.** Prefrontal layer 5 pyramidal neurons recorded from littermate WT, *Grin1*KD and *Grin1*rescue mice. All mice received tamoxifen treatment in adulthood and were recorded subsequent to washout. Data shown as mean ± SEM.

**Supplemental Figures**


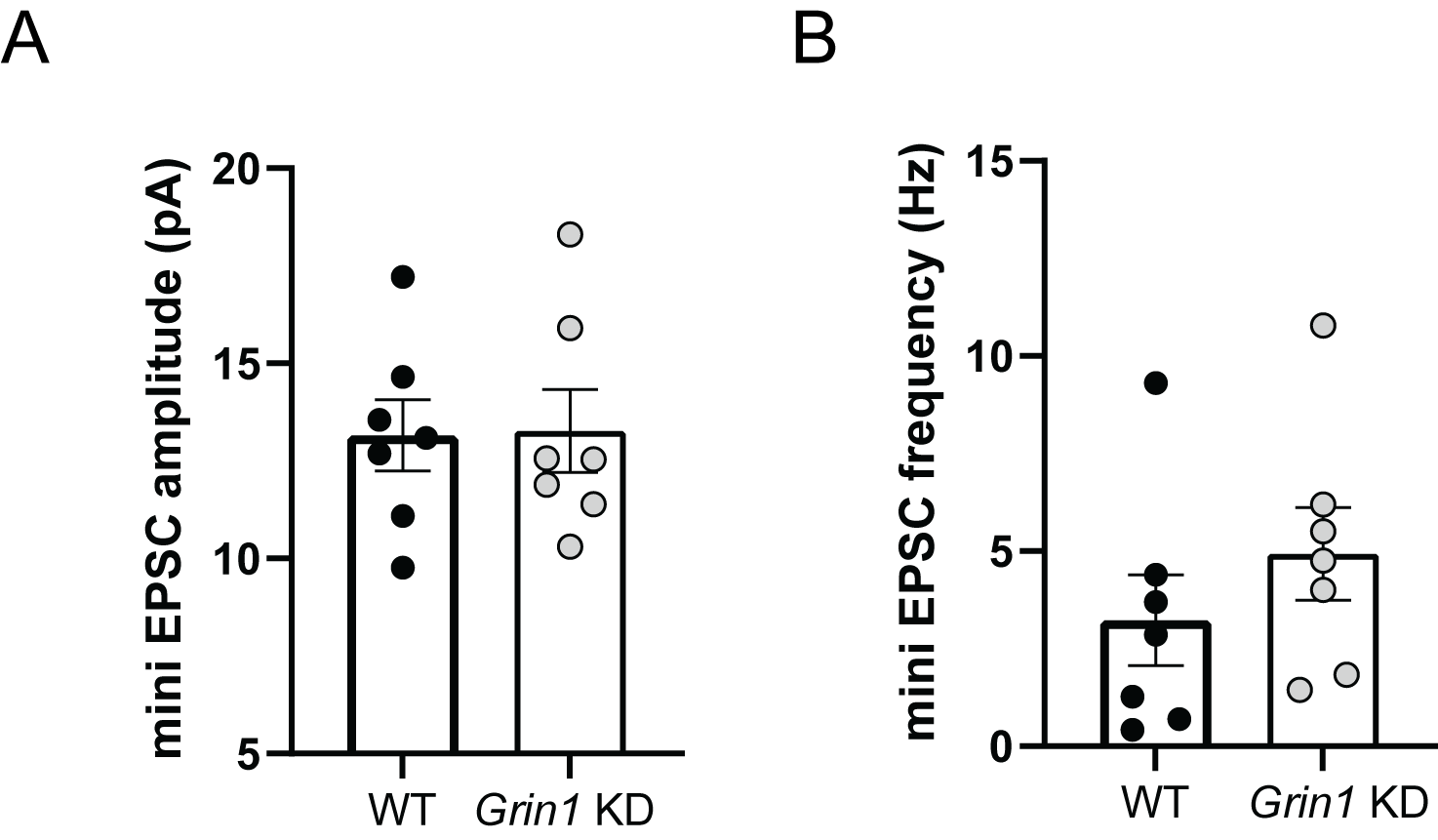


**Supplemental Figure S1.**

**Miniature excitatory postsynaptic currents (mini EPSCs) do not differ between WT and *Grin1*KD.** Measured in the presence of tetrodotoxin (TTX), mini EPSCs are similar in (**A**) amplitude (t_12_ = 0.08, *P* = 0.93) and (**B**) frequency (t_12_ = 1.02, *P* = 0.33) between WT and *Grin1*KD.


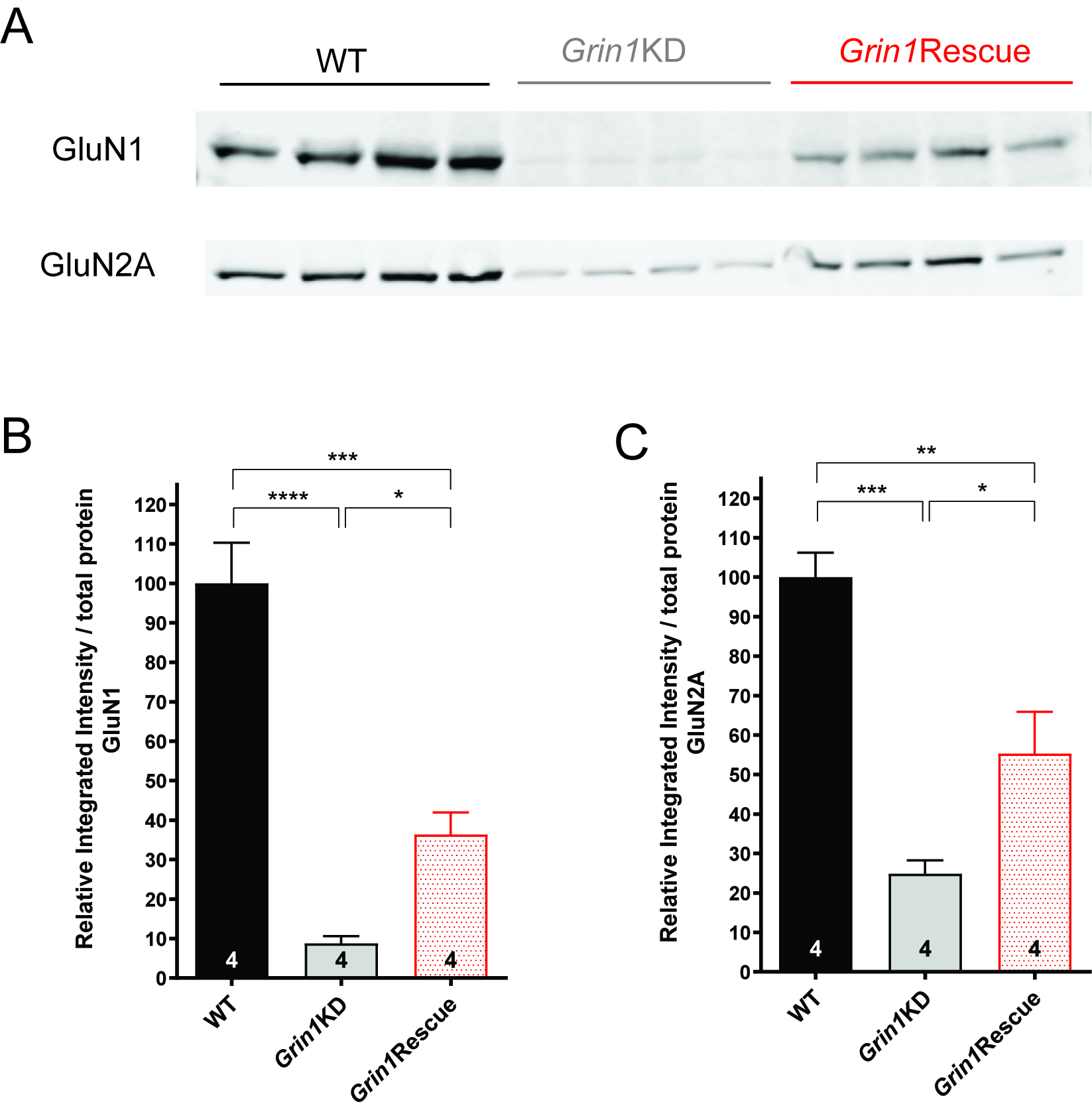


**Supplemental Figure S2.**

**GluN1 and GluN2A subunit protein levels are decreased in *Grin1*KD, and increase in *Grin1*Rescue mice following tamoxifen treatment.** (**A**) Immunoblot of 30µg crude synaptoneurosomal protein extract from cortex in male and female WT, *Grin1*KD, and *Grin1*Rescue mice aged 14-weeks (tamoxifen intervention at 10-weeks). *Grin1*KD mice show a marked decrease in the level of (**B**) GluN1 protein expressed, and subsequently a decrease in (**C**) GluN2A levels as well. *Grin1*Rescue mice show an increase in GluN1 and GluN2A subunit protein levels following tamoxifen treatment. Total protein stain was used as loading control. Data shown as mean ± SEM. N values are as indicated in the figure; **P* < 0.05, ***P* < 0.01, ****P* < 0.001, *****P* < 0.0001, Multiple comparisons one-way ANOVA with post-hoc Tukey’s test.


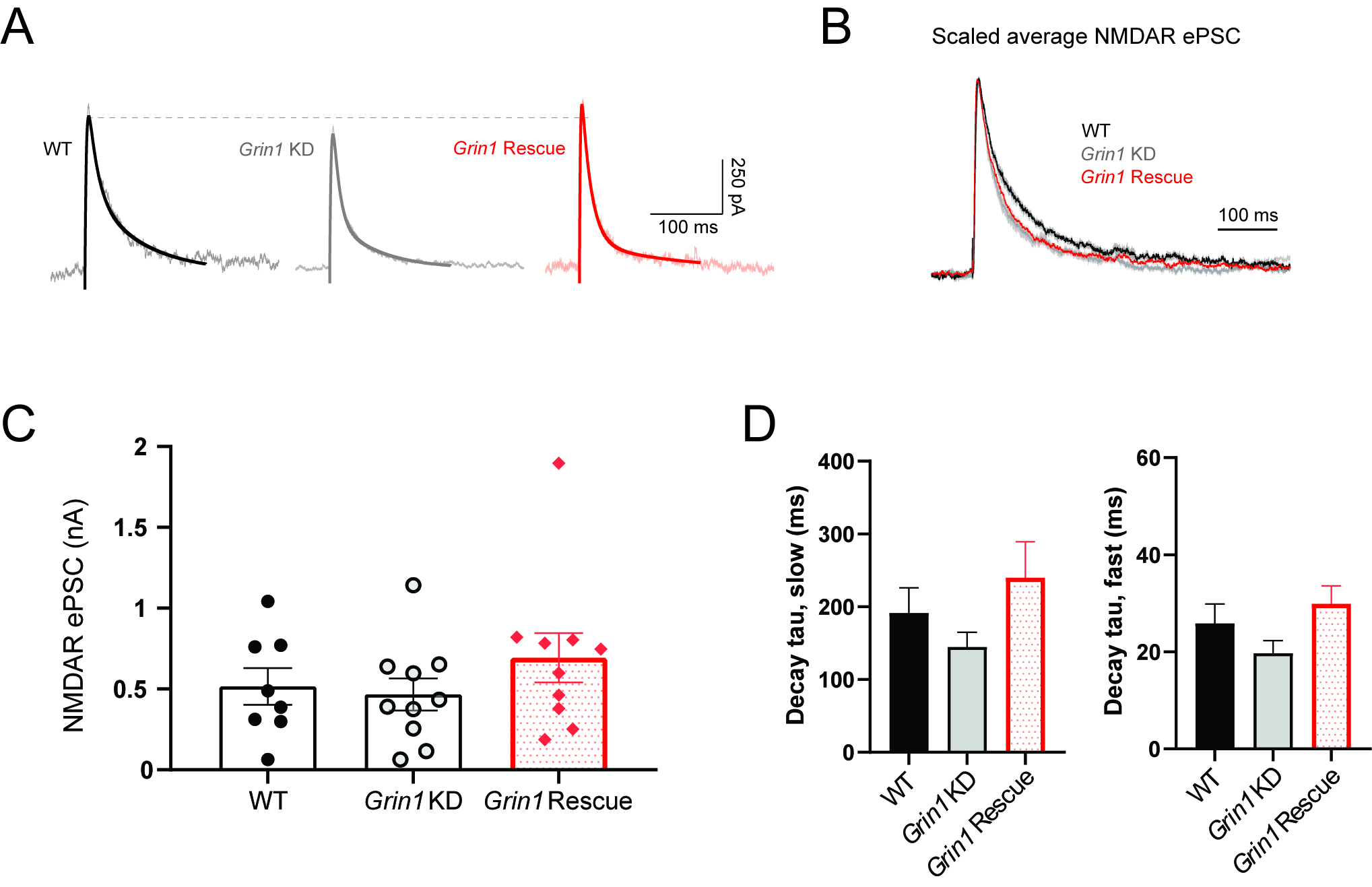


**Supplemental Figure S3.**

**NMDAR ePSCs evoked by minimal stimuli show similar amplitude and kinetics in WT, *Grin1*KD and *Grin1*Rescue.** (**A**), Examples of NMDAR evoked postsynaptic currents (ePSCs) in tamoxifen treated WT, *Grin1*KD and *Grin1*Rescue are shown with double exponential fit. (**B**) Scaled NMDAR ePSC average of three genotypes. (**C**) Amplitude of evoked NMDAR ePSC is not significantly different across the three genotypes (F_2, 25_ = 0.95, *P* = 0.40) similar to figure 1. (**D**) Decay kinetics of NMDAR ePSC are not significantly different across genotypes. Slow decay tau (F_2, 23_ = 1.74, *P* = 0.19) and fast decay tau (F_2, 23_ = 2.28, *P* = 0.12) show no effect of genotype.

**Supplemental Methods**

***Electrophysiological Recordings***

Mice were anesthetized with chloral hydrate (400 mg/kg) and sacrificed, with brains removed and quickly placed in ice-cold sucrose ACSF solution. Coronal brain slices (400 µM) of the prefrontal cortex were cut with the Dosaka Pro-7 Linear Slicer and transferred to a recovery chamber containing ACSF at 30 C, bubbled with 95 % oxygen and 5 % carbon dioxide. After at least 2-hr recovery, slices were transferred to a modified chamber mounted on the stage of an Olympus microscope and submerged in oxygenated ACSF at 30°C.

***Solutions***

Sucrose ACSF (in mM):

254 sucrose, 10 D-glucose, 26 NaHCO_3_, 2 CaCl_2_, 2 MgSO_4_, 3 KCl, 1.25 NaH_2_PO_4_

Normal ACSF (in mM):

128 NaCl, 10 D-glucose, 26 NaHCO_3_, 2 CaCl_2_, 2 MgSO_4_, 3 KCl, 1.25 NaH_2_PO_4_

Reduced Mg2+ ACSF (in mM):

128 NaCl, 10 D-glucose, 26 NaHCO_3_, 2 CaCl_2_, 0.5 MgSO_4_, 5 KCl, 1.25 NaH_2_PO_4_

Regular pipette solution (in mM):

120 K-gluconate, 5 KCl, 2 MgCl_2_, 4 K_2_-ATP, 0.4 Na_2_-GTP, 10 Na_2_-phosphocreatine, 10 HEPES buffer with the final pH adjusted to 7.3 with KOH

Pipette solution for voltage-clamp measurements of NMDAR currents at positive holding potentials (in mM):

105 Cs–gluconate, 17.5 CsCl, 10 Cs_4_-BAPTA, 2 Mg-ATP, 0.3 Na_2_-GTP, 5 QX-314 bromide, 10 HEPES buffer, and final pH adjusted to 7.3 with CsOH

***Pharmacology***

Pharmacological agents were applied with the oxygenated ACSF in specific experiments: 6-cyano-7-nitroquinoxaline-2,3-dione (CNQX), picrotoxin, CGP52432, D-2-amino-5-phosphopentanoic acid (D-APV), NMDA. All compounds were from Sigma-Aldrich, Tocris, or Alomone Labs. The compounds were stored in stock solution at -20 °C until diluted in oxygenated ACSF.

***Grin1Rescue mice***

*Grin1*KD mice with loxP sites flanking an insertion Neo cassette were crossed with Cre-ERT2 mice and the adult offspring were treated with tamoxifen (**Fig 4A**). In *Grin1*KD mice with the Cre-ERT2 transgene, tamoxifen induces Cre-mediated excision of the Neo cassette in *Grin1*, restoring full-length mRNA expression and NMDAR levels to ~60% of wild-type (Mielnik et al., Molecular Psychiatry, 2021).

***Immunoblotting***

We assessed cerebral cortex protein levels of GluN1 and GluN2A subunits in 12 WT, *Grin1KD*, and *Grin1*rescue mice of both sexes. The mice were treated at age 10 weeks with tamoxifen for 2 weeks and sacrificed at 14 weeks. Cortical tissue was dissected from brains frozen in cold isopentane (over dry ice). Tissue homogenates of synaptoneurosomes were prepared, as previously described (Li et al., Science, 2010). Tissue was homogenized in homogenization solution (0.32M sucrose, 20mM HEPES pH 7.4, 1mM EDTA) for 30-sec with a hand-held motorized pestle. At 4°C, homogenate was spun at 1000G for 10-min, and then supernatant spun at 10000G 10-min. Pellet was resuspended in lysis buffer (50mM Tris HCl pH 7.5, 150mM NaCl, 1% Triton X-100, 0.1% SDS, 2mM EDTA). Protein concentration was measured using BCA assay (Thermo Scientific). 30µg of protein were resolved on 4% stacking and 8% separating gels and transferred to PVDF membranes (Pall Life Sciences, NY, USA) via 1-4hour transfer at 100V. Total protein was stained for using REVERTTM Total Protein Stain Kit (LI-COR, Lincoln, NE, USA). Membranes were blocked in 5% milk in TBS-T (TBS + 0.1% (v/v) Tween-20) for 30-min and then incubated in primary antibodies (5% milk in TBS-T) overnight at 4°C. Primary antibodies for GluN1 (NR1) and GluN2A (NR2A) proteins were as follows: NR1 – 1:250, mouse IgG, Upstate (Millipore) Cat No: 05-432, Lot: 2538; NR2A – 1:1000, rabbit IgG, Upstate (Millipore) Cat No: 07-632, Lot: 32704. Blots were washed in TBS-T, incubated with anti-mouse IRDye 680 (1:10000, LI-COR) and anti-rabbit IRDye 800 (1:10000, LI-COR). Blots were visualized and densitometry was analyzed using the LI-COR Odyssey system and software.
